# Dl-3-n-Butylphthalide Rescues Dopaminergic Neurons in Parkinson’s Disease Models by Inhibiting the NLRP3 Inflammasome and Ameliorating Mitochondrial Impairment

**DOI:** 10.3389/fimmu.2021.794770

**Published:** 2021-12-01

**Authors:** Rongfang Que, Jialing Zheng, Zihan Chang, Wenjie Zhang, Hualing Li, Zhenchao Xie, Zifeng Huang, Hai-Tao Wang, Jiangping Xu, Dana Jin, Wanlin Yang, Eng-King Tan, Qing Wang

**Affiliations:** ^1^ Department of Neurology, Zhujiang Hospital, Southern Medical University, Guangzhou, China; ^2^ School of Pharmaceutical Sciences, Southern Medical University, Guangzhou, China; ^3^ College of Biological Sciences, University of California, Davis, Davis, CA, United States; ^4^ Department of Neurology, National Neuroscience Institute, Singapore General Hospital, Singapore, Singapore; ^5^ Department of Neurology, Duke-National University of Singapore Medical School, Singapore, Singapore; ^6^ Department of Neurology, The Third Affiliated Hospital of Sun Yat-Sen University, Sun Yat-Sen University, Guangzhou, China

**Keywords:** Parkinson’s disease, dl-3-n-Butylphthalide, NLRP3 inflammasome, neuroinflammation, α-Synuclein, PARP1, mitochondrial dysfunction, neurovascular unit

## Abstract

**Background:**

Neuroinflammation and mitochondrial impairment play important roles in the neuropathogenesis of Parkinson’s disease (PD). The activation of NLRP3 inflammasome and the accumulation of α-synuclein (α-Syn) are strictly correlated to neuroinflammation. Therefore, the regulation of NLRP3 inflammasome activation and α-Syn aggregation might have therapeutic potential. It has been indicated that Dl-3-n-butylphthalide (NBP) produces neuroprotection against some neurological diseases such as ischemic stroke. We here intended to explore whether NBP suppressed NLRP3 inflammasome activation and reduced α-Syn aggregation, thus protecting dopaminergic neurons against neuroinflammation.

**Methods:**

In our study, we established a MPTP-induced mouse model and 6-OHDA-induced SH-SY5Y cell model to examine the neuroprotective actions of NBP. We then performed behavioral tests to examine motor dysfunction in MPTP-exposed mice after NBP treatment. Western blotting, immunofluorescence staining, flow cytometry and RT-qPCR were conducted to investigate the expression of NLRP3 inflammasomes, neuroinflammatory cytokines, PARP1, p-α-Syn, and markers of microgliosis and astrogliosis.

**Results:**

The results showed that NBP exerts a neuroprotective effect on experimental PD models. *In vivo*, NBP ameliorated behavioral impairments and reduced dopaminergic neuron loss in MPTP-induced mice. *In vitro*, treatment of SH-SY5Y cells with 6-OHDA (100uM,24 h) significantly decreased cell viability, increased intracellular ROS production, and induced apoptosis, while pretreatment with 5uM NBP could alleviated 6-OHDA-induced cytotoxicity, ROS production and cell apoptosis to some extent. Importantly, both *in vivo* and *in vitro*, NBP suppressed the activation of the NLRP3 inflammasome and the aggregation of α-Syn, thus inhibited neuroinflammation ameliorated mitochondrial impairments.

**Conclusions:**

In summary, NBP rescued dopaminergic neurons by reducing NLRP3 inflammasome activation and ameliorating mitochondrial impairments and increases in p-α-Syn levels. This current study may provide novel neuroprotective mechanisms of NBP as a potential therapeutic agent.

## Highlights

1. NBP attenuates dopaminergic neurotoxicity and ameliorates motor impairments in MPTP-induced mouse models.
2. NBP protects SH-SY5Y cells from 6-OHDA-induced injury.
3. NBP inhibits the activation of NLRP3 inflammasome and prevents the activation of microglia and astrocytes, thus suppresses the inflammatory response in PD models.
4. NBP reduces the abnormal aggregation of p-a-Syn and ameliorated the mitochondrial impairment in PD models.

## Introduction

Parkinson’s disease (PD) is the second neurodegenerative disorders. From a pathological perspective, the characteristics of PD are a gradual loss of dopaminergic neurons in the substantia nigra (SN) and an aggregation of α-synuclein (α-Syn) (1, 2). To date, the treatment for PD remains symptomatic, and no effective therapies to slow down or prevent the disease processes (3). The development of effective treatments that not only modify the disease but also delay disease progression are urgently needed.

Studies by our group and other researchers have suggested that neuroinflammation is of considerable importance in the pathologic process of PD (4–12). In PD, neuroinflammatory cells are composed of hyperplastic reactive glial cells, especially microglia and astrocytes, the essential components of the neurovascular unit (NVU) (13). The over-activation of microglia and the release of proinflammatory cytokines play important roles in neurodegeneration in individuals with PD (14). Several lines of evidence have indicated microglia activation in the SN regions of PD animal models and patients with PD, along with a widespread increase in the NLRP3 inflammasome (15). The NLRP3 inflammasome is a cytoplasmic protein complex that contains NLRP3, ASC and caspase-1. Its activation enhances the release of interleukin-1β (IL-1β) and interleukin-18 (IL-18), and display a critical role in the pathogenesis and development of inflammation and immune response (16, 17). In central nervous system, some researchers have confirmed that NLRP3 inflammasome is mainly localized in microglia, while some studies have shown that NLRP3 also exists in neurons (18, 19). Notably, the activation of NLRP3 inflammasome has been proved to be involved in the pathogenesis of many neurodegenerative diseases, including AD and PD (20–22). The results of Lee et al. suggest that NLRP3 inflammasome played an important role in the neurotoxicity and the loss of dopaminergic neurons in MPTP-treated mice and cell culture models (23). Impaired autophagy of microglia aggravates MPTP-induced neurodegeneration by regulating NLRP3 inflammasome (24). Some studies have demonstrated the intimate positive association between NLRP3 inflammasome-mediated dopaminergic neuronal injury induced by the microglial activation and the accumulation of α-Syn in PD (25). In PD models, Icaritin was confirmed to stabilize mitochondrial function, thus inhibiting the activation of NLRP3 inflammasome and reducing neuroinflammation (26); and Salvianolic acid B has also been shown to reduce neuronal injury and inhibit inflammation by negatively regulating the expression of NLRP3 (27). Therefore, inhibiting the activation of NLRP3 inflammasomes may be a significant strategy to delay PD progression, and further study is needed to verify this hypothesis.

Mitochondrial dysfunction is another important factor in the damage of dopaminergic neurons in Parkinson’s disease. More and more evidence show that α-Syn is associated with mitochondrial dysfunction in PD (28). α-Syn is a useful biomarker for diagnosing Parkinson’s disease and for detecting the disease progression (29, 30), and some researchers have attempted to find out new medications that may reduce the concentration of α-Syn in the brain to prevent its aggregation and sequestration in LBs (31). According to recent studies, α-Syn presents important roles in regulating nonspecific immunity and is closely related to the neuroinflammatory response (32, 33). Poly (ADP-ribose) polymerase-1 (PARP1) is a ribozyme that regulates intracellular homeostasis and genomic stability. The activity of PARP1 increases when it detects minor DNA damage, and then the poly(ADP-ribose) (PAR) polymer is synthesized to activate the DNA base excision repair system. However, when DNA damage shifts from mild to severe, PARP1 becomes overactivated to produce large amounts of PAR, leading to cell death (34). Neurotoxic α-Syn aggregates have been shown to induce PARP1 overactivation. Kam et al. reported that α-Syn fibrils activate PARP1 and then trigger PAR generation, while PARP1 and PAR subsequently directly bind to and accelerate the fibrillization of recombinant α-Syn into highly pathogenic fibrils. In other words, in the pathological state, a vicious cycle will be formed between α-Syn, PARP1, and PAR, which is called a “feed-forward loop” (35). Therefore, the association between PARP1 and α-Syn might lead to the progress of PD therapeutic strategies.

Dl-3-n-butylphthalide (NBP), has been approved for the therapy of acute ischemic stroke in patients since 2002 in China (36–38). Recently, studies have shown that NBP holds extensive pharmacological activities (39–45) and may exert potentially therapeutically beneficial effects on ischemia and vascular dementia; however, its precise mechanisms remain unclear. Evidences have suggested that NBP treatment could exert neuroprotective actions by enhancing anti-oxidation and attenuating mitochondrial dysfunction in models of ischemic stroke and could inhibit NLRP3 inflammasome activation *via* upregulating Nrf2 in AD models (45, 46). As NBP may provide neuroprotection by attenuating mitochondrial impairment and neuroinflammation, we aimed to explore whether NBP induces neuroprotection in PD models by modulating the NLRP3 inflammasome and how NBP interacts with α-Syn by influencing mitochondrial impairment. This study will provide a promising potential therapeutic approach for PD neuropathogenesis.

## Materials and Methods

### Main Regents and Kits

NBP was generously provided by Shijiazhuang Pharma Group NBP Pharmaceutical Co., Ltd, (Shijiazhuang, China). 1-Methyl-4-phenyl-1,2,3,6-tetrahydropyridine (MPTP) and 6-OHDA were purchased from Sigma–Aldrich (St. Louis, MO, USA). Cell Counting Kit-8 (CCK-8) and Reactive Oxygen Species (ROS) Assay kits were obtained from Beyotime Biotechnology Co., Ltd. (Nan Tong, China). Annexin V-FITC Apoptosis Detection Kit and Hoechst 33258 dye were acquired from KeyGen Biotech Co., Ltd. (Nanjing, China). The BCA protein assay reagent and the RIPA lysis buffer were obtained from Thermo Fisher Scientific (MA, USA). Anti-tyrosine hydroxylase (TH, ab137869 and ab113), anti-alpha-synuclein (α-Syn, ab138501), anti-alpha-synuclein (phospho S129) (p-α-Syn, ab51253), anti-poly [ADP-ribose] polymerase 1 (PARP1, ab191217), anti-allograft inflammatory factor 1 (IBA1, ab178847) antibodies, donkey anti-rabbit IgG (Alexa Fluor 488) (ab150073), donkey anti-rabbit IgG (Alexa Fluor 647) (ab150075) and donkey anti-sheep IgG (Alexa Fluor 647) (ab150179) antibodies were purchased from Abcam (Cambridge, USA). Anti-apoptosis-associated speck-like protein containing a CARD (ASC, 67824), anti-poly/mono-ADP ribose (PAR, 83732) and anti-phospho-histone H2AX (Ser139) (p-γH2AX, 9718) antibodies were bought from Cell Signaling Technology, Inc. (Beverly, USA). Anti-NACHT, LRR and PYD domain-containing protein 3 (NLRP3, 19771-1-AP), anti-Caspase 1/p20/p10 (Caspase 1, 22915-1-AP), anti-Interleukin-1 beta (IL-1β, 16806-1-AP), anti-glial fibrillary acidic protein (GFAP, 16825-1-AP), anti-beta actin (β-actin, 60008-1-Ig), goat anti rabbit IgG (SA00001-2), and goat anti-mouse IgG (SA00001-1) antibodies were purchased from Proteintech Group (Chicago, IL, USA).

### Animals

**Figure 1:**
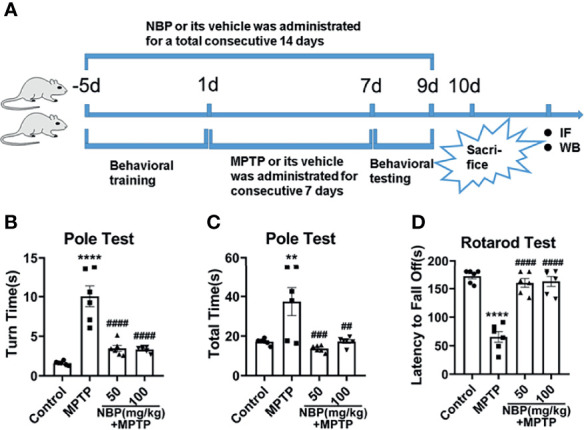
Diagram of the experimental design and behavioral tests in MPTP-induced mice. **(A)** Experimental design and drug administration scheme to explore the role and mechanism of NBP in MPTP-induced PD mouse model. Total time **(B)** and turn time **(C)** spent in the pole test. **(D)** Latency to fall in the rotarod test. All data are presented as mean ± SEM (n = 6). **p < 0.01, ****p < 0.0001, compared with the Control group; ^##^p < 0.01, ^###^p < 0.001, ^####^p < 0.0001, compared with the MPTP group.

All animal experiments were preapproved by the Experimental Animal Ethics Committee of Zhujiang Hospital of Southern Medical University (Approval No. LAEC-2020-228). Male C57BL/6 mice (8-week-old, 25-30g) were obtained from Guangdong Experimental Animal Center. Before the experiment, the animals were domesticated for 7 days in standard facilities. Drug concentrations were chosen based on previous reports. Some previous studies give us a clue on employing the experimental concentration of NBP (43, 47). Combined with our pilot study, 50 mg/kg (low) and 100 mg/kg (high), the two comprehensive considerations were applied in the present study. Forty male mice were completely divided into 4 groups at random as followed (1): control, (2) MPTP, (3) MPTP+NBP (50 mg/kg), and (4) MPTP+NBP (100 mg/kg). Mice in the MPTP group were intraperitoneally injected with MPTP (30 mg/kg) for 5 days, and the same amount of vegetable oil as used in the treatment group was administered five days before the MPTP injection for 14 days consecutively. Mice in the treatment group were injected with NBP (50 mg/kg or 100 mg/kg, dissolved in vegetable oil) five days before the intraperitoneal injection of MPTP (30 mg/kg, dissolved in saline) for 14 consecutive days. At the same time, saline was intraperitoneally injected in control mice. All mice were sacrificed after performing behavioral tests to obtain brain tissues (**Figure 1A**).

### Rotarod Test

Motor coordination in mice was evaluated through the rotarod test. Before the establishment of the model, all mice received adaptive training on the rotarod until no falls were observed within 5 minutes. All the mice walked on rod as it accelerated from 5rpm to 40rpm within 90 seconds and maintained 40rpm for 180s. Then, we recorded the longest time each mouse stayed on the rod.

### Pole Test

We assessed movement disorders by performing pole tests. Similarly, before modeling, we trained the mouse to climb down smoothly from the top of the pole (height 55 cm, diameter 1 cm) to the bottom. The time required for the mouse to turn and climb down the pole was recorded.

### Immunofluorescence Staining

The mice were anesthetized with sodium pentobarbital and sacrificed, perfused with normal saline, and then fixed with 4% paraformaldehyde. The brain was dissected and stored frozen in 30% sucrose for 48 hours, and then we made coronal sections of the frozen brains. For immunofluorescence staining, brain slices were incubated with an anti-GFAP antibody (1:500) and anti-IBA1 antibody (1:300) to observe the proliferation of glial cells and incubated with anti-TH antibody (ab113, 1:800) to detect the level of dopaminergic neuron damage. Then, slices were incubated with donkey anti-rabbit IgG (Alexa Fluor 488) and/or donkey anti-sheep IgG (Alexa Fluor 647) secondary antibodies, and images were captured using a fluorescence microscope.

### Cell Culture and Treatment

The culture medium of SH-SY5Y was high glucose DMEM supplemented with 10% fetal bovine serum and 1% penicillin/streptomycin/glutamine and cells were cultured in a humid environment at 37°C with 5% CO2. According to the purpose of the experiment, the cells were divided into four groups: control, 6-OHDA, NBP and 6-OHDA+NBP. The concentrations of 6-OHDA and NBP were determined based on the cell viability assay.

### Determination of Cell Viability

We clarified the damaging effect of 6-OHDA and the protective effect of NBP on SH-SY5Y cells by performing a cell viability test. First, cells were exposed to certain concentrations of 6-OHDA (50, 100, or 200 µM) for 24 hours, the effect of 6-OHDA on cell viability was observed. Second, cells were processed with various concentrations of NBP solution (0.1, 1, 10, or 100 µM) alone for 30 hours, and the effects of NBP solution on cell viability were observed. Finally, the cells were pretreated with different concentrations of NBP solution (0.1, 1, 5, 10, or 100 µM) for 6 hours and then exposed to 100 µM 6-OHDA for 24 hours. The effect of NBP on the viability of cells induced by 6-OHDA was observed, and the appropriate concentration of NBP was selected. The CCK-8 calorimetric method was performed to detect cell viability. In brief, the seeding density of cells was 5×103 cells per well in 96-well plate. At the end of culture, 10 microliters of CCK-8 buffer were added and incubated at 37°C for about 2 hours. Finally, cell viability was measured at 450 nm absorbance.

### Apoptosis Analysis

Apoptosis Detection Kit was used to explore the rate of apoptotic cells in each group through flow cytometry. Cells in the 6-OHDA+NBP group were pretreated with 5 µM NBP for 6 hours and then 100 µM 6-OHDA was added; and the cultivation was continued for a whole day. Cells were collected and washed, and then double staining with PI and Annexin V-FITC in binding buffer was conducted. Finally, apoptosis was detected using a Cytoflex flow cytometer. In addition, apoptosis was detected using Hoechst staining. SH-SY5Y cells were contained with Hoechst 33258 (1 ug/ml) within 10 minutes. The morphological changes in the stained nuclei were observed to identify apoptotic cells.

### Measurement of Reactive Oxygen Species

Dichlorodihydrofluorescein diacetate (DCFH-DA) fluorescence was measured to assess the level of intracellular reactive oxygen species (ROS) in each group. Briefly, cells were incubated with DMEM containing 10 µM DCFH-DA for half an hour and then washed with PBS buffer 3 times. After trypsin digestion, cells were centrifuged at 1000 rpm for 5 minutes and then re-suspended in 200 µL of serum-free DMEM. Flow cytometry was performed to observe the fluorescence intensity of cells in each group, and images of DCFH-DA were captured under a fluorescence microscope.

### Measurement of Cytokine Levels

Total mRNA was extracted, and then mRNAs were reverse transcribed into cDNAs. Finally, real-time quantitative PCR was performed to explore the mRNA levels of several representative inflammatory cytokines in cells from each group, including tumor necrosis factor-α (TNF-α), interleukin-4 (IL-4), and interleukin-6 (IL-6). The sequences of the specific primers for the target gene are listed below.

TNF-α: forward(5’-TGGCGTGGAGCTGAGAGATAACC-3’) and reverse (5’-CGATGCGGCTGATGGTGTGG-3’);

IL-4: forward(5’-AAAACTTTGAACAGCCTCACAG-3’) and reverse (5’-GGTTTCCTTCTCAGTTGTGTTC-3’);

IL-6: forward(5’-CACTGGTCTTTTGGAGTTTGAG-3’) and reverse (5’-GGACTTTTGTACTCATCTGCAC-3’);

GAPDH: forward(5’-ACCCAGAAGACTGTGGATGG-3’) and reverse (5’- TTCAGCTCAGGGATGACCTT-3’).

### Western Blotting Analysis

Cells were collected and lysed with RIPA lysis buffer, and the supernatant was collected by centrifugation, and then the total protein concentration was detected by BCA protein assay. The extracted protein (40ug per sample) was transferred to PVDF membrane after SDS-polyacrylamide gel electrophoresis. Membranes were blocked with 5% skim milk solution at 37°C for about 1-4 hours, and then incubated with the primary antibodies all night along at 4°C: anti-TH (ab137869, 1:1000), anti-Nurr1 (1:3000), anti-NLRP3 (1:1000), anti-ASC (1:1000), anti-caspase-1 (1:500), anti-IL-1β (1:500), anti-α-Syn (1:1000), anti-p-α-Syn (1:500), anti-PARP1 (1:1000) and anti-PAR (1:1000). Finally, specific secondary antibodies were added for incubation with the membranes for one hour. The control was anti-β-actin (1:5000). Blots were identified with an ECL system and analyzed with ImageJ software. The protein band intensity was normalized to β-actin and indicated as the ratio of the control.

### Cellular Immunofluorescence Staining

For cellular immunofluorescence (IF) staining, we first fixed the cells for 30 minutes and next permeabilized them for 15-30 minutes and then blocked them at 37°C for an hour and finally incubated them with the primary antibodies overnight: rabbit anti-NLRP3 (1:100), rabbit anti-ASC (1:100), and sheep anti-TH (ab113, 1:800). The second day, the cells were treated with secondary antibodies bound to fluorophores for an hour in dark. For double-labeled immunofluorescence, the cells were incubated overnight with the first primary antibody followed by the other primary antibody on the next day, and finally incubated with the matching secondary antibody at 37°C for an hour on the third day. The cells were retained with the nuclear dye for 5 minutes. Nikon microscope was used to capture representative fluorescence images, and the fluorescence intensity was analyzed using ImageJ software.

### Statistical Analysis

All results were analyzed using GraphPad Prism 8.01 (GraphPad Software), and data were shown as mean ± standard error of the mean (SEM). One-way analysis of variance (ANOVA) with Tukey’s test for *post hoc* comparisons was used to define the differences between groups. Data are representative of at least three independent experiments. A p-value<0.05 was considered statistically significant.

## Results

### NBP Ameliorates Behavioral Impairments in MPTP-Induced Mice

We induced PD-like symptoms and analyzed the impact of NBP on the motor function of PD mice. According to the results of the pole test and rotarod test, MPTP administration resulted in a significant impairment of motor function, and NBP treatment ameliorated this impairment. It took notably more time for MPTP-induced mice to turn around and climb down in the pole test (**Figure 1B, C** **)** and less time on the rotarod test (**Figure 1D**). However, compared with MPTP mice, MPTP+NBP-treated mice displayed considerably improved performance on the pole test (**Figures 1B, C**) and rotarod test (**Figure 1D**).

### NBP Attenuates Dopaminergic Neurotoxicity in MPTP-Induced Mice

**Figure 2:**
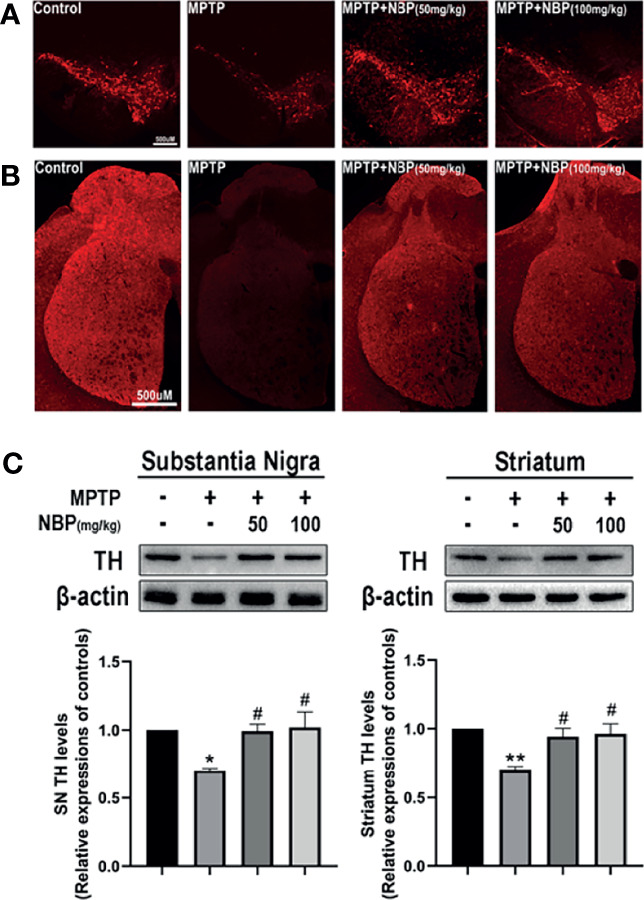
NBP confers protection in dopaminergic neurons in the MPTP-induced model of Parkinson’s disease. **(A)** Representative immunofluorescence images of tyrosine hydroxylase staining in the substantia nigra pars compacta (SN) of each group. **(B)** Representative immunofluorescence images of axon fibers in the mouse striatum. **(C)** Representative immunoblot images and summarized data for TH levels in the substantia nigra and striatum. All data are presented as means ± SEM (n = 3). *p < 0.05 and **p < 0.01 compared with the control group; ^#^p < 0.05 compared with the MPTP group.

We performed immunofluorescence staining to observe the loss of dopaminergic neurons in the nigrostriatal system after MPTP and NBP administration, and determined the effect of NBP on TH protein expression using Western blot. The immunofluorescence staining results showed that MPTP-induced mice showed a serious loss of TH-positive neurons and fibers in the SN (**Figure 2A**) and striatum (**Figure 2B**). Compared with the MPTP group, TH were considerably increased in the MPTP+NBP group (**Figures 2A, B** **)**. These were further endorsed by Western blots, which showed higher TH protein levels in the SN and striatum of NBP-treated mice (**Figure 2C**). NBP reversed the decrease of TH induced by MPTP and dopaminergic neurotoxicity in PD mice.

### NBP Inhibits MPTP-Induced Microgliosis and Astrogliosis

**Figure 3:**
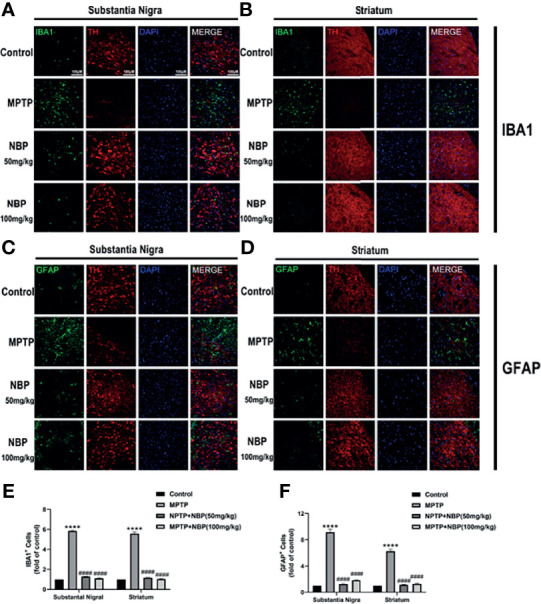
NBP Inhibits MPTP-induced Microgliosis and Astrogliosis. **(A, B, E)** Immunofluorescence staining for IBA1-positive microglia (green), TH (red), and DAPI (blue) in the SN and striatum showing that NBP significantly decreased MPTP-induced microglial activation. **(C, D, F)** Immunofluorescence staining for GFAP-positive astrocytes (green), TH (red), and DAPI (blue) in the SN and striatum showing that NBP significantly decreased MPTP-induced astrocyte activation. All data are presented as means ± SEM (n=3). ****p < 0.0001 compared with the control group; ^####^p < 0.0001 compared with the MPTP group.

The MPTP-induced proliferation of microglia and astrocytes in rodents is a well-known pathological feature. Therefore, we evaluated whether NBP regulated glial cell proliferation induced by MPTP injection *in vivo*. Immunofluorescence staining for the microglial marker IBA1 demonstrated a meaningfully increased number of activated microglia in the SN and striatum of the MPTP group. Importantly, this MPTP-induced proliferation of microglia decreased in the MPTP+NBP group (**Figures 3A, B, E** **)**. Similarly, immunofluorescence staining for the astrocyte marker GFAP confirmed significantly greater astrocyte proliferation in the SN and striatum of the MPTP group than that of the control group; but astrocyte proliferation in the MPTP+NBP group was greatly inhibited compared with the MPTP group (**Figures 3C, D, F** **)**. Taken together, NBP may attenuate dopaminergic degeneration in PD mice by reducing the proliferation of microglia and astrocytes.

### NBP Ameliorates NLRP3-Associated Neuroinflammation in MPTP-Induced Mice

**Figure 4:**
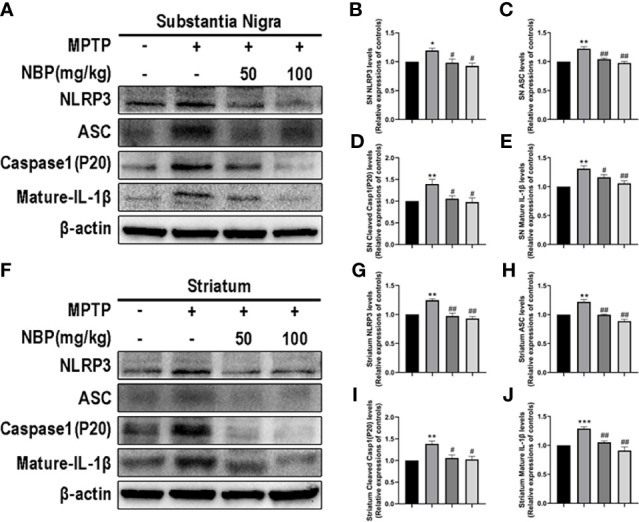
NBP Ameliorates NLRP3-associated Neuroinflammation in the nigrostriatal system. **(A)** Representative Western blot bands and graphs showing the statistical analysis of NLRP3 **(B)**, ASC **(C)**, cleaved Caspase1 **(D)** and mature IL-1β **(E)** levels in the SN. **(F)** Representative Western blot bands and the graphs showing the statistical analysis of NLRP3 **(G)**, ASC **(H)**, cleaved Caspase1 **(I)** and mature IL-1β **(J)** levels in the striatum. All data are presented as means ± SEM (n = 3). *p < 0.05, **p < 0.01, and ***p < 0.001 compared with the control group; ^#^p < 0.05 and ^##^p < 0.01 compared with the MPTP group.

The NLRP3 inflammasome expression in the brain tissue of mice in each group was detected by Western blotting to explore the effects of NBP on NLRP3 inflammasome *in vivo*. Levels of the NLRP3 (**Figures 4A, B, F, G** **)** and ASC (**Figures 4A, C, F, H** **)** proteins were considerably increased in the SN and striatum of the MPTP-induced mice. At the same time, MPTP also induced significant activation of caspase-1 (P20) (**Figures 4A, D, F, I** **)** and increased levels of the mature IL-1β protein (**Figures 4A, E, F, J** **)**. Importantly, NBP treatment significantly reduced the protein levels of NLRP3, ASC, cleaved Caspase1, and mature IL-1β (**Figure 4**), thus significantly inhibiting the activation of the NLRP3 inflammasome. MPTP induces a substantial improvement in the expression of NLRP3 inflammasomes, while NBP reduces the expression of NLRP3 inflammasomes, suggesting that NBP may reduce MPTP-induced neuronal damage by inhibiting NLRP3 inflammasomes.

### NBP Provides Neuroprotection by Attenuating Mitochondrial Impairment *In Vivo*

**Figure 5:**
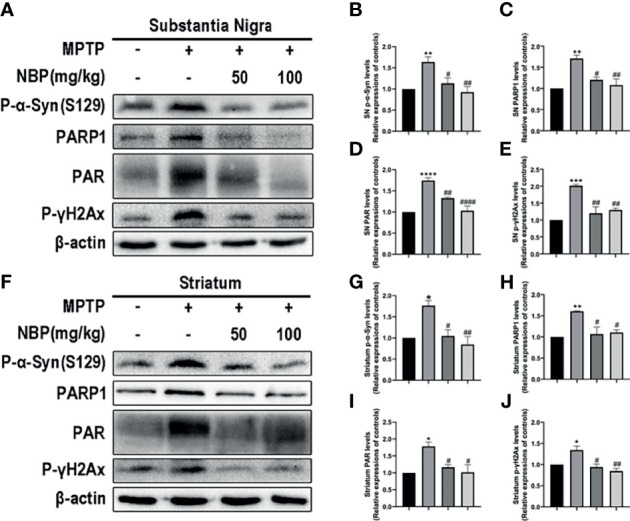
NBP provides neuroprotection by attenuating mitochondrial impairments. **(A, F)** Levels of the p-α-Syn, PARP1, PAR and p-γH2Ax proteins in the SN and striatum were detected by Western blot assay. **(B–E, G–J)** The ratios of densitometry values for p-α-Syn, PARP1, PAR and p-γH2Ax to β-actin in the SN and striatum were calculated and normalized to each respective control culture. All data are presented as means ± SEM (n = 3). *p < 0.05, **p < 0.01, ***p < 0.001, and ****p < 0.0001 compared with the control group; ^#^p < 0.05, ^##^p < 0.01, and ^####^p < 0.0001 compared with the MPTP group.

After determining the effect of NBP on NLRP3 inflammasome *in vivo*, we next explored whether NBP ameliorated the MPTP-induced mitochondrial impairment. Levels of the α-Syn, PARP1 and PAR proteins in both the SN and striatum were evaluated using Western blotting. MPTP induced significantly increased levels of p-α-Syn in both the SN and striatum as against the control group, while the treatment of NBP significantly reduced the MPTP-induced increases in p-α-Syn levels in the SN and striatum compared with the MPTP group (**Figures 5A, B, F, G** **)**. In addition, MPTP also increased the levels of PARP1, PAR and p-γH2Ax in the SN and striatum, and NBP attenuated the effects of MPTP on PARP1, PAR and p-γH2Ax expression (**Figure 5**). These findings imply that NBP inhibits PARP1 activation and the aggregation of p-α-syn *in vivo*, thus ameliorating mitochondrial impairments.

### NBP Protects SH-SY5Y Cells From 6-OHDA-Induced Injury

**Figure 6:**
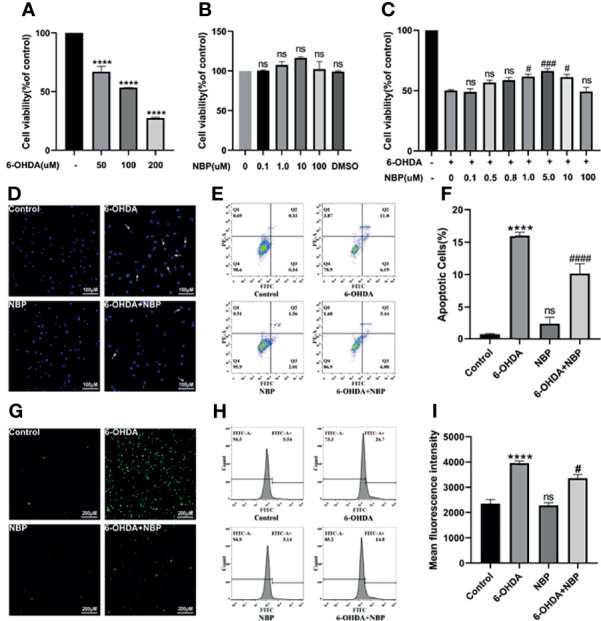
NBP Protects SH-SY5Y Cells From 6-OHDA-induced Injury. **(A)** Cell viability was determined 24 h after induction with different concentrations of 6-OHDA. **(B)** The viability of SH-SY5Y cells incubated with the indicated concentrations of NBP for 30 h **(C)** Cells were pretreated with NBP for 6 h before exposure to 100 µM 6-OHDA for an additional 24 h **(D)** The effects of NBP on apoptotic cells were determined using Hoechst 33258 staining. **(E)** The effect of NBP on cell apoptosis was also measured using Annexin-V/PI staining, and the apoptotic rate calculated from the flow cytometry data is shown **(F)**. Intracellular ROS levels were measured using DCFH-DA staining with immunofluorescence detection **(G)** and flow cytometry **(H)**, and the fluorescence intensity data are also shown **(I)**. All data are presented as the means ± SEM (n = 3). ****p < 0.0001, compared with the control group; ^#^p < 0.05, ^###^p < 0.001, and ^####^p < 0.0001 compared with the 6-OHDA group; ns, not significant, compared with the control group in **(A, B, F, I)**, and compared with the 6-OHDA group in **(C)**.

Meanwhile, we explored the effect of NBP on a PD cell model and further confirmed the potential protective role of NBP in cell death. First, when the cells were treated with 6-OHDA (100uM, 24h), cell viability was decreased to approximately 50% (**Figure 6A**). Next, we evaluated the role of NBP itself on cell viability under normal culture conditions. The results showed that 0.1‒100 µM NBP had no effect on the viability of SH-SY5Y cells, and DMSO, the solvent of NBP (the highest concentration was less than 0.02%), had no effect on the growth of cells (**Figure 6B**). Finally, we explored the effect of NBP on the viability of SH-SY5Y cells induced by 6-OHDA. NBP exerted a dose-dependent protective effect on cell viability and 5uM treatment increased the viability of SH-SY5Y cells more effectively than other concentrations **(** **Figure 6C**). Therefore, we chose to use this concentration in the next experiment. After that, we performed PI/Annexin V-FITC staining and Hoechst 33258 staining to analyze apoptosis. We found that cell apoptosis was remarkably worsen in the 6-OHDA group, but the NBP pretreatment (6-OHDA+NBP group) significantly reduced 6-OHDA-induced apoptosis (**Figures 6D–F** **)**. Based on these results, to a certain extent, NBP improves cell viability and plays a protective role in SH-SY5Y cells induced by 6-OHDA.

### NBP Suppresses ROS Generation in 6-OHDA-Stimulated SH-SY5Y Cells

Oxidative stress is presumed to activate the NLRP3 inflammasome, so we speculate that the effect of NBP on inhibiting the NLRP3 inflammasome is associated with the reduction in ROS levels. The detection of DCFH-DA fluorescence demonstrated that intracellular ROS levels were significantly increased after 6-OHDA treatment, while NBP pretreatment considerably decreased 6-OHDA-induced ROS production (**Figures 6G–I** **)**. Therefore, the NBP pretreatment comparatively prevents the ROS production induced by 6-OHDA.

### NBP Increases Tyrosine Hydroxylase Expression in 6-OHDA-Induced SH-SY5Y Cells

**Figure 7:**
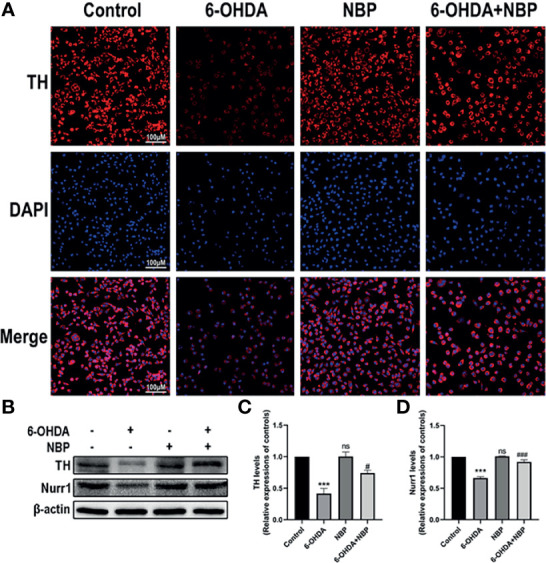
Neuroprotective effects of NBP on TH and Nurr1 expression in 6-OHDA-induced SH-SY5Y cells. **(A)** 6-OHDA-induced SH-SY5Y cell damage was evaluated by immunocytofluorescence staining. **(B)** The protein expression of TH and Nurr1 was detected by western blot assay. **(C, D)** the statistical graph of TH and Nurr1 in SH-SY5Y cell models. All data are presented as mean ± SEM (n = 3). ***p < 0.001, ns, not significant, compared with the Control group; ^#^p < 0.05, ^###^p < 0.001, compared with the 6-OHDA group.

TH is an important enzyme in dopamine synthesis. We detected the effect of NBP on TH levels in the SH-SY5Y cell model using cellular immunofluorescence (**Figure 7A**) and Western blotting (**Figures 7B–D** **)** to further confirm the neuroprotective effect of NBP. As the results showed in **Figure 7**, TH decreased significantly after 100 µM 6-OHDA induction for 24 hours, and this effect was reversed by 5 µM NBP pretreatment, indicating that NBP treatment has a protective effect on dopaminergic neuronal damage induced by 6-OHDA.

### NBP Inhibits 6-OHDA-Induced NLRP3 Inflammasome Activation *In Vitro*

**Figure 8:**
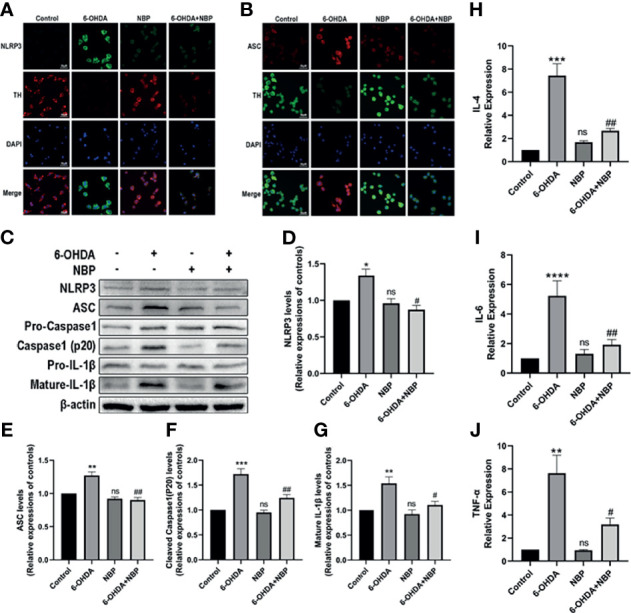
NBP inhibits NLRP3 inflammasome activation and decreases the levels of inflammatory cytokines induced by 6-OHDA *in vitro*. **(A)** Immunofluorescence detection of NLRP3 (green) in SH-SY5Y cells. **(B)** Immunofluorescence detection of ASCs (red) in SH-SY5Y cells. **(C)** Levels of the NLRP3, ASC, pro-caspase-1, cleaved caspase-1, pro-IL-1β and mature IL-β proteins were detected using Western blot assays. **(D–G)** The ratios of densitometry values of NLRP3, ASC, cleaved caspase-1 and mature IL-1β to β-actin were calculated and normalized to each respective control culture. **(H–J)** Real-time RT–PCR analysis showing the effects of NBP on IL-4, IL-6, and TNF-α after 6-OHDA injury. All data are presented as the means ± SEM (n=3). *p < 0.05, **p < 0.01, ***p < 0.001, ****p < 0.0001, and ns, not significant, compared with the control group; ^#^p < 0.05 and ^##^p < 0.01 compared with the 6-OHDA group.

We next verified the role of NBP on the NLRP3 inflammasome in PD models by performing immunofluorescence staining and Western blotting to examine the expression of the NLRP3 inflammasome in cell models after different treatments. We observed the expression levels of NLRP3 and ASC in using immunofluorescence staining. 6-OHDA induced a meaningful rise in the fluorescence intensity of NLRP3 and ASC, while 6-OHDA+NBP significantly reduced their fluorescence intensity (**Figures 8A, B** **)**. These results are similar to the Western blot which showed increased levels of the NLRP3, ASC, cleaved Caspase1 and mature IL-1β proteins in SH-SY5Y cells exposed to 6-OHDA; while the pretreatment of NBP significantly reduced the expression of these proteins (**Figures 8C–G**). Based on these results, NBP exerts an inhibitory effect on NLRP3 inflammasomes *in vitro*. In addition, we explored the role of NBP on proinflammatory cytokines by performing RT–qPCR to detect the mRNA levels of IL-4, IL-6 and TNF-α in cells. The results showed that the levels of IL-4, IL-6 and TNF-α in the 6-OHDA group were considerably increased compared with the control group, while the levels in the 6-OHDA+NBP group were significantly decreased (**Figure 8H–J**).

### NBP Protects SH-SY5Y Cells From the 6-OHDA-Induced Mitochondrial Impairment

**Figure 9:**
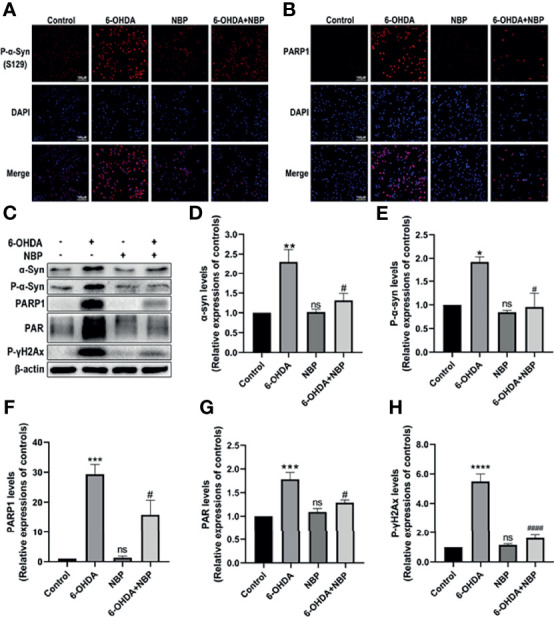
NBP protects SH-SY5Y cells from 6-OHDA-induced mitochondrial impairment. **(A)** Immunofluorescence detection of phosphorylated α-synuclein (p-α-Syn, S129) (red) in SH-SY5Y cells. **(B)** Immunofluorescence detection of PARP1 (red) in SH-SY5Y cells. **(C)** Levels of the α-synuclein, p-α-Syn, PARP1, PAR and phosphorylated γ-H2Ax (p-γH2Ax) proteins were detected using Western blot assays. **(D-H)** The ratios of densitometry values for α-synuclein, p-α-Syn, PARP1, PAR and p-γH2Ax to β-actin were calculated and normalized to each respective control culture. All data are presented as the means ± SEM (n = 3). *p < 0.05, **p < 0.01, ***p < 0.001, ****p < 0.0001, and ns, not significant, compared with the control group; ^#^p < 0.05 and ^####^p < 0.0001 compared with the 6-OHDA group.

After identifying the effect of NBP on mitochondrial impairments *in vivo*, we then explored whether NBP exerted a similar effect on PD cell models *in vitro*. We addressed this question by examining the levels of p-α-Syn (**Figure 9A**) and PARP1 (**Figure 9B**) in SH-SY5Y cells using immunofluorescence staining, and Western blotting was performed to evaluate levels of the p-α-Syn, α-Syn, PARP1, PAR and p-γH2AX proteins (**Figures 9C–H**). As shown in **Figure 9**, 6-OHDA induced α-Syn aggregation and phosphorylation, and this aggregated and phosphorylated state of α-Syn was alleviated by NBP Similarly, NBP inhibited the activation of PARP1, PAR and p-γH2AX. Taken together, these data suggest that NBP might attenuate mitochondrial impairments by reducing PARP1, PAR and p-γH2AX levels.

## Discussion

**Figure 10:**
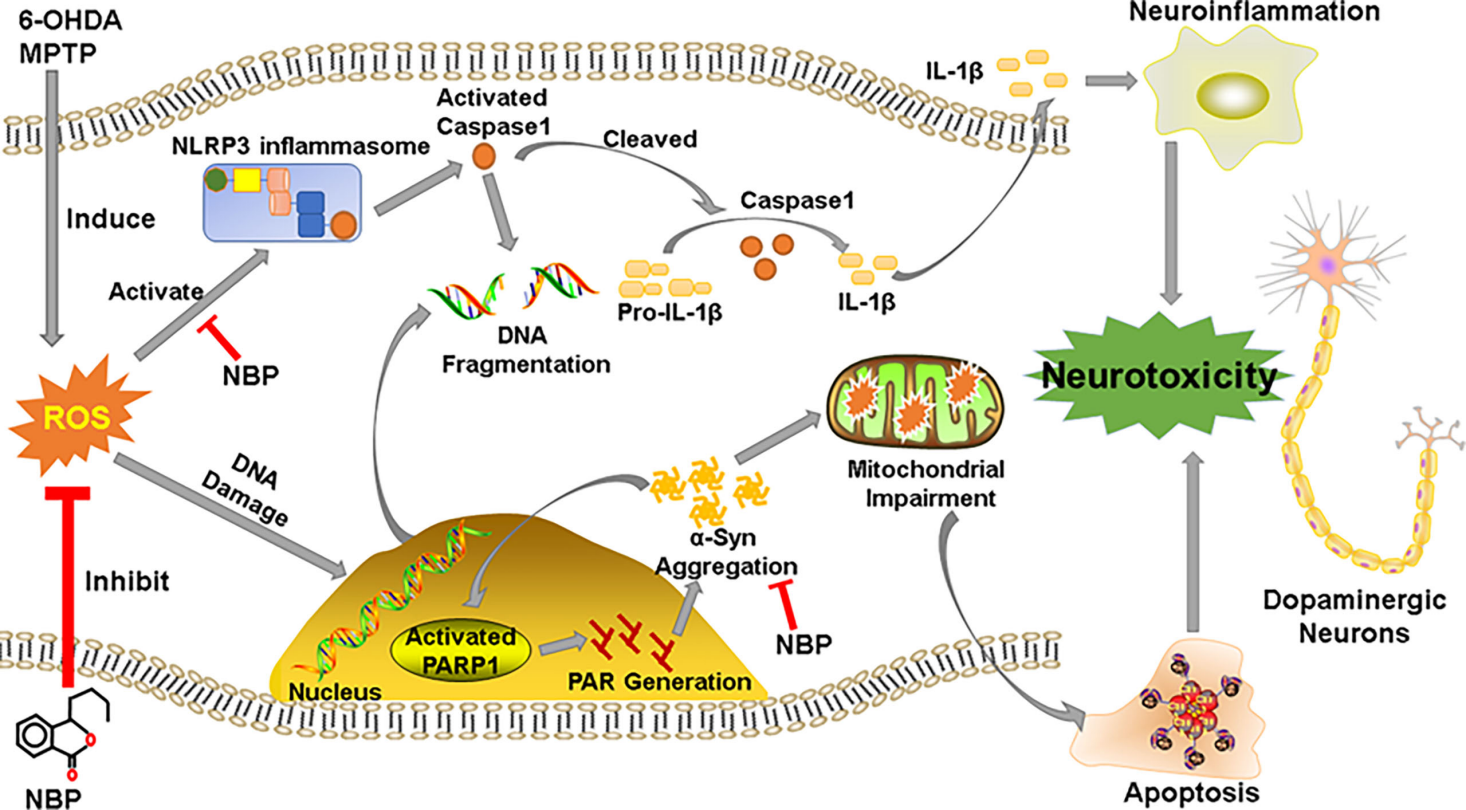
Schematic representation of the possible mechanism for the neuroprotective effect of NBP in PD. See the *Discussion* part.

Our study shows that NBP protected dopaminergic neurons from 6-OHDA- or MPTP-induced neurotoxicity. We described three main findings. First, NBP could alleviated the loss of dopaminergic neuronal in the SN and striatum, improving motor dysfunctions in MPTP-induced PD mice; and pretreatment with NBP could improve cell viability and reduced cellular apoptosis and ROS production in 6-OHDA-induced cellular model. Second, we observed that NBP attenuated neuroinflammation by inhibiting the activation of the NLRP3 inflammasome, preventing the activation of microglia and astrocytes and the upregulated expression of inflammatory factors. Finally, NBP reduced the abnormal aggregation of p-a-Syn and ameliorated mitochondrial impairments in PD models. This current study may provide a novel neuroprotective mechanism of NBP as a useful therapeutic agent for PD (**Figure 10**).

Some neurodegenerative diseases are related to inflammation, including AD and PD (14, 48). Previous studies support the importance of neuroinflammatory mechanisms in the pathogenesis of PD (30, 49–52). Accompanying the occurrence and development of PD, neuroinflammation accelerates PD progression by aggravating the degeneration and loss of dopaminergic neurons (53, 54). Clinical research has explored the role of nonsteroidal anti-inflammatory drugs (NSAIDs) as treatments for patients with PD, and some epidemiological results have shown that NSAIDs might reduce the risk of PD (55–57), although conflicting outcomes have also been reported. Therefore, an understanding of the mechanisms of neuroinflammation and clarifying its effects on the occurrence and development of PD present interesting challenges. Although more and more evidences show that neuroinflammation based on the activation of microglia is one of the important pathological features of Parkinson’s disease, the exact mechanism of neuroinflammation is not completely clear, and the immune function of neuron itself is also controversial (21). Furthermore, studies have shown that, in addition to immune function, microglia also play a vital role in supporting the formation of neuronal synapses in the brain, and can monitor the changes in neuronal activity (58). The communication between microglia and neurons is the basis for the normal function of the central nervous system. The interruption of microglia-neuron communication can lead to harmful processes, such as neuroinflammation and neurodegeneration (59). In this study, NBP exerted a protective role on MPTP-induced neurotoxicity in mice and 6-OHDA-induced SH-SY5Y cells, and reduced the release of IL-4, IL-6 and TNF-α, suggesting that the neuroprotective effects of NBP are probability related to the inhibition of neuroinflammation (**Figures 3**, **8**).

The inflammasome, which is a complex composed of numerous proteins, is an important part of the inflammatory response, and NLRP3 is the most important inflammasome. It is a multiprotein complex consisting of NLRP3, ASC and Caspase1 that regulates the activation of IL-1β and IL-18 and plays an important role in neuroinflammation-related diseases (60). Over the last few years, the role of the NLRP3 inflammasome in the central nervous system has received increasing attention. NLRP3 inflammasome is mainly localized in microglia, while some studies have demonstrated that NLRP3 is also present in neurons. Fann et al. detected the NLRP3 protein in primary cortical neurons stimulated by glucose in mouse (61); Zou et al. detected it in human hippocampal neurons of postmortem after alcoholism (62); Ye et al. found that more NLRP3 were co-localized with the neuronal marker NeuN than the microglia marker CD68, and they suggested that the activation of microglia may be related to the activation of NLRP3 inflammasome in neurons (63). Recently, the NLRP3 inflammasome was reported to be activated in the brains of individuals with PD and in the microglia of a PD mouse model, suggesting that the NLRP3 inflammasome participates in the neuropathogenesis of PD (23, 64). There seems to be a complicated relationship between NLRP3 inflammasome in neurons and microglia, jointly promoting neuroinflammation, which needs to be further studied in the future.

Besides, oxidative stress was presumed to activate the NLRP3 inflammasome and contribute to neuroinflammation. Studies have suggested that increased intracellular ROS accumulation can promote NLRP3:ASC:pro-caspase-1 complex assembly and enhance microglia activation, while using antioxidant to reduce ROS production can suppress NLRP3 inflammasome and microglia activation, and finally alleviate microglia mediated neurotoxicity (65, 66). This is consistent with our results which showed that NBP could reduce the ROS generation and inhibit the neuroinflammation. The NLRP3 inflammasome may be activated by the abnormal α-Syn aggregates and oxidative stress in the brains of patients with PD (67), while the NLRP3, mature IL-1β and cleaved Caspase-1 protein levels are increased in a rodent model of PD (68). Moreover, the absence of the NLRP3 inflammasome in PD mice may reduce motor dysfunction, as well as dopaminergic neurodegeneration. As a consequence, inhibiting the activation of the NLRP3 inflammasome may be a beneficial interference for PD (23, 69). Consistent with these findings, our current results showed significantly increased IL-4, IL-6 and TNF-α in 6-OHDA-induced cells, and the NLRP3 inflammasome-related proteins in MPTP-induced mice and 6-OHDA-induced cells were also significantly increased, strongly indicating that the NLRP3 inflammasome is activated in the PD *in vivo* and vitro models. However, after pretreatment with NBP, the increased expression of inflammatory cytokines and NLRP3 inflammasome-related proteins was significantly abolished, indicating that NBP suppressed the activation of the NLRP3 inflammasome. Thus, NBP might play an anti-inflammatory role by reducing the activation of the NLRP3 inflammasome and further prevent neuronal degeneration by attenuating the inflammatory response in subjects with PD. These findings not only identify NBP as a promising therapeutic drug for PD but also support the reduction of NLRP3 inflammasome as a possible target for the therapy of neurodegenerative diseases.

The aggregation of abnormal α-Syn in neurons is a remarkable pathological characteristic of PD. Neuroinflammation promotes the formation of α-Syn aggregates in neurons (33), and the α-Syn polymer is considered a potential activator of NLRP3 inflammasomes in patients with PD (25). Treatments targeting α-Syn have good potential in clinical applications in PD (7). In terms of PARP1 and PAR, there is evidence that overactivated PARP1 and aggregated PAR are involved in the impairment of mitochondrial function (70). Recent studies have confirmed that PARP1 is significantly activated in PD models and that the level of PAR is increased in the cerebrospinal fluid (CSF) of PD individuals (71). In addition, a recent study observed the interactions of α-Syn/PAPR1/PAR in post mortem PD/PDD patient samples (72). According to Kam et al., PARP1 activation and PAR formation are the main mediators of pathological α-Syn toxicity and transmission (35). Thus, the inhibition of PARP1 and reduction in PAR levels might reduce the neurotoxicity caused by pathological α-Syn transmission. Interestingly, our study detected activated PARP1 and phosphorylated α-Syn in PD models, while the administration of NBP also inhibited PARP1 activation and downregulated p-α-Syn levels, subsequently ameliorating neuronal apoptosis. Our results imply that the therapeutic effects of NBP are probably mediated by the “α-Syn/PARP1/PAR” feed-forward loop (35).

In recent years, accumulating evidence has shown that neuroinflammation is a significant mechanism underlying the occurrence and development of PD. Inhibition of neuroinflammation reduces the dopaminergic neurons loss in patients with PD and slows the progression of PD. Therefore, anti-inflammatory drug therapy may be an important breakthrough in the drug treatment of PD. NBP exerts anti-inflammatory effects on various neurological disease models. A recent study of ischemic mice showed that NBP inhibits the activation of vascular inflammation, thus maintaining the integrity of the blood–brain barrier and microvascular patency; this study provided evidence that NBP reduces ischemic brain injury by inhibiting neurovascular inflammation (39). In addition, NBP administration has been demonstrated to regulate neuroinflammatory processes and attenuate neuronal damage in mouse models of amyotrophic lateral sclerosis (73). NBP attenuates Aβ‐induced inflammation in cultured astrocytes *in vitro*. Furthermore, in the study by Wang and colleagues, NBP inhibited the activation of the NLRP3 inflammasome by intervening the TXNIP-NLRP3 interaction, thereby reducing neuroinflammation and neurodegeneration (45). On the other hand, Tschopp et al. proposed that mitochondria are involved in the activation of NLRP3 inflammasome (74). Damaged mitochondria could over-activate the NLRP3 inflammasome, thus promoting the secretion of proinflammatory cytokines (75). However, the over-activation of NLRP3 inflammasome in turn would aggravate mitochondrial damage (76). Therefore, reducing mitochondrial impairments show a stronger inhibitory effect on NLRP3 inflammasome-related neuroinflammation. Our current results show that NBP not only inhibited the activation of the NLRP3 inflammasome and reduced the level of inflammatory cytokines, but also reduced the abnormal accumulation and phosphorylation of α-Syn in animal and cell models of PD by ameliorating mitochondrial impairments, thus reducing the pathological changes in α-Syn, which in turn also reduced the inflammatory reaction to some extent. Thus, NBP may be an effective and potential anti-inflammatory alternative for the treatment of PD. However, our research and findings have certain limitations. For example, we still do not know the exact mechanism underlying the association between NLRP3 inflammasome-mediated neuroinflammation and α-Syn aggregate-triggered neuroinflammation, and the interaction of NLRP3 and α-Syn is not clear. In addition, our findings are derived from experimental animals and cell models, and the translation of potential effective drugs from animal and cellular research to clinical trials requires extensive effort; thus, follow-up studies in humans must be investigated.

## Conclusions

In this study, *in vivo* and *in vitro* PD models were used to identify that NBP ameliorates mitochondrial impairments, prevents the activation of the NLRP3 inflammasome and reduces anti-inflammatory responses. This study provides a convincing theoretical basis for the potentially clinical application of NBP in PD. In general, regardless of the origin of the neuroinflammatory process of PD, treatments aimed at preventing or downregulating these immune-related mechanisms may be very beneficial in delaying disease progression or even preventing the pathological process.

## Data Availability Statement

The raw data supporting the conclusions of this article will be made available by the authors, without undue reservation.

## Ethics Statement

The animal study was reviewed and approved by the Experimental Animal Ethics Committee of Zhujiang Hospital of Southern Medical University.

## Author Contributions

RQ, E-KT, and QW conceived and designed the study. RQ, JZ, ZC, WZ, HL and ZX performed the study. ZH, WY, H-TW, DJ, and JX revised the article for intellectual content. RQ and JZ performed data statistics and analysis. RQ, WY, E-KT, and QW wrote the article. All authors contributed to the article and approved the submitted version.

## Funding

This work was supported by the National Key R&D Program of China (Grant NO: 2017YFC1310200), National Natural Science Foundation of China (NO: 81873777, 82071414), Initiated Foundation of Zhujiang Hospital (NO: 02020318005), and Science and Technology Program of Guangdong of China (NO: 2020A0505100037) to QW; National Natural Science Foundation of China (NO: 82001343) to WY.

## Conflict of Interest

The authors declare that the research was conducted in the absence of any commercial or financial relationships that could be construed as a potential conflict of interest.

## Publisher’s Note

All claims expressed in this article are solely those of the authors and do not necessarily represent those of their affiliated organizations, or those of the publisher, the editors and the reviewers. Any product that may be evaluated in this article, or claim that may be made by its manufacturer, is not guaranteed or endorsed by the publisher.

## Abbreviations

AD, Alzheimer’s disease; α-Syn, alpha-Synuclein; GFAP, Glial fibrillary acidic protein; IBA1, Allograft inflammatory factor 1; IL-1β, interleukin-1β; IL-4, interleukin-4; IL-6, interleukin-6; IL-18, interleukin-18; LBs, Lewy bodies; MPTP, 1-methyl-4-phenyl-1,2,3,6-tetrahydropyridine; NBP, Dl-3-n- butylphthalide; ROS, reactive oxygen species; PD, Parkinson’s disease; PAR, Poly (ADP-ribose); PARP1, Poly (ADP-ribose) polymerase-1; SN, substantia nigra; TH, Tyrosine Hydroxylase; TNF-α, tumor necrosis factor-α; 6-OHDA, 6-hydroxydopamine.
